# Postnatal causes and severity of persistent pulmonary Hypertension of Newborn

**DOI:** 10.12669/pjms.37.5.2218

**Published:** 2021

**Authors:** Muhammad Sohail Arshad, Mudasser Adnan, Hafiz Muhammad Anwar-ul-Haq, Arif Zulqarnain

**Affiliations:** 1 Dr. Muhammad Sohail Arshad, FCPS (Paeds Cardiology) Department of Paediatric Cardiology, The Children’s Hospital & the Institute of Child Health, Multan, Pakistan; 2 Dr. Mudasser Adnan, FCPS (Paeds Medicine) Department of Paediatric Cardiology, The Children’s Hospital & the Institute of Child Health, Multan, Pakistan; 3 Dr. Hafiz Muhammad Anwar-ul-Haq, FCPS (Paeds Medicine) Department of Paediatric Cardiology, The Children’s Hospital & the Institute of Child Health, Multan, Pakistan; 4 Dr. Arif Zulqarnain, FCPS (Paeds Medicine) Department of Paediatric Cardiology, The Children’s Hospital & the Institute of Child Health, Multan, Pakistan

**Keywords:** Birth asphyxia, Meconium aspiration syndrome, Newborn, Persistent pulmonary hypertension, Respiratory distress syndrome

## Abstract

**Background & Objective:**

Persistent pulmonary hypertension of the newborn (PPHN) is described as severe respiratory failure along with hypoxaemia. PPHN is known to be linked with high morbidity and mortality around the world. This study was planned to determine the postnatal causes and assess the severity of persistent pulmonary hypertension of newborn in babies presenting to the Children’s Hospital, Multan.

**Methods:**

This observational study was conducted at the Department of Paediatric Cardiology, The Children Hospital &Institute of Child Health, Multan, Pakistan from July to December 2019. A total of 122 confirmed cases of PPHN admitted having gestational age above 34 weeks were enrolled. Demographic data of the newborns was recorded along with maternal medical history, pregnancy status and postnatal causes of PPHN. Severity of PPHN was also recorded.

**Results:**

Out of a total of 122 cases of PPHN, 81 (66.3%) were male. Majority, 78 (64.0%) had gestational age above 37 weeks. Mode of delivery as cesarean section was noted in 70 (57.4%). Meconium aspiration syndrome 52 (42.6%), birth asphyxia 48 (39.3%), respiratory distress syndrome 23 (18.8%) and sepsis 33 (27.0%) were found to be the commonest causes of PPHN. Severe PPHN was found to be the most frequent, noted among 63 (51.6%) while Moderate PPHN was observed in 40 (32.8%) and Mild PPHN in 19 (15.6%). Morality was noted among 26 (21.3%) of cases.

**Conclusion:**

Meconium aspiration syndrome, birth asphyxia and respiratory distress syndrome were the commonest postnatal causes of PPHN. Severe PPHN was found to be the most frequent form of PPHN.

## INTRODUCTION

Persistent pulmonary hypertension of the newborn (PPHN) is described as severe respiratory failure along with hypoxaemia.^1^ PPHN is known to be linked with high morbidity and mortality around the world. Incidence of PPHN is calculated as 2/1000 live births while mortality ranging 4-33% has been seen in developed countries.^2^,^3^ Recently published data from six Asian countries found the incidence of PPHN ranging 1.2 to 4.6 per 1000 live births whereas overall mortality as 20.1% among cases of PPHN was observed.^4^ Numbers of cases with adverse outcomes are pronounced in developing countries.^5^,^6^ In the last few decades, significant progress has been made tackling PPHN but it still poses major threat.

Development of PPHN is attributed to failure of circulatory transition at the time of birth that causes pulmonary artery pressure to be higher in comparison to systemic pressure.^7^ Severe hypoxaemia is caused because of a patent ductus arteriosus (PDA) or the right to left shunting of blood through a patent foramen ovale (PFO).^8^,^9^ PPHN has been noted to affect term or near terms while preterm neonates have also been seen to get affected.

PPHN was earlier labeled as persistent fetal circulation but later, the current term came into use being since it better describes the underlying pathophysiology. Commonly, PPHN is followed to an underlying pulmonary pathology while primary of PPHN having unknown origin also exists.^10^ Meconium aspiration syndrome (MAS), idiopathic PPHN, respiratory distress syndrome, pneumonia or sepsis are known to be some of the major causes of PPHN.^3^,^9^ Local data suggests male babies, cesarean section as mode of delivery, use of positive pressure ventilation while resuscitation and birth asphyxia as some of the commonest risk factors associated with PPHN.^5^

When major difference between preductal and postductal oxygen saturation is seen along with severe hypoxaemia not improving despite 100% supplemental oxygen, PPHN is suspected. Confirmation of PPHN diagnosis is made on the basis of echocardiography as clinically, it is not easy to distinguish PPHN from cyanotic congenital heart disease (CHD).^3^,^11^

Scarcity of local data exists regarding PPHN. Children’s Hospital, Multan is the largest neonatal tertiary care hospital of South Punjab, Pakistan. This study was planned to determine the postnatal causes and to assess the severity of persistent pulmonary hypertension of newborn in babies presenting to the Children’s Hospital, Multan.

## METHODS

This observational study was conducted at the Department of Paediatric Cardiology, The Children Hospital &Institute of Child Health, Multan, Pakistan from July to December 2019. Approval from Institutional Ethical Committee (Ref. No. ERC 168/19) was taken for this. Informed consent was also sought for all the study participants.

A total of 122 confirmed cases of PPHN admitted having gestational age above 34 weeks (as per mother’s last menstrual period) were enrolled. Cases with congenital cyanotic heart disease or chromosomal anomalies were excluded.

Diagnosis of PPHN was made by an experienced Paediatric Cardiologist on the basis of presentation with refractory hypoxaemia along with one or more than one of these:^4^

(i) echocardiographic evidence of elevated pulmonary pressure described as right-to-left or bidirectional shunt at PDA and/or PFO level.
(ii) pre-to-postductal partial pressure of oxygen gradient (PaO2) ≥ 20 mmHg.
(iii) a pre-to-postductal pulse oximetry oxygen saturation (SpO2) gradient ≥ 5%.
(iv) positive hyperoxia-hyperventilation test. Newborns having cyanotic CHD or chromosomal anomalies were not enrolled.

Demographic data of the newborns along with maternal medical history and pregnancy conditions, fetal distress, need of resuscitation and evidence of birth asphyxia along with other causes (e.g., MAS, RDS, birth asphyxia, neonatal sepsis, congenital pneumonia, transient tachypnea of the newborn, lung hypoplasia, idiopathic PPHN) and severity of PPHN were recorded on a predesigned proforma. Once confirmed as a case of PPHN, newborn was identified as mild, moderate or severe PPHN. Severity of PPHN was made on the basis of tricuspid regurgitation (TR) measurement as indicated by echocardiography.^5^ TR between 40-50 mmHg was labeled as mild PPHN, 50 to 70 mmHg as moderate PPHN while TR above 70 mmHg as severe PPHN.^5^ All newborns were managed as per our unit’s management protocol made for PPHN. SPSS version 20.0 was used for the purpose of data analysis. Data was represented in terms of frequency and percentages.

## RESULTS

The basic characteristics of studied cases with PPHN is shown in Table-I. Out of a total of 122 cases of PPHN, 81 (66.3%) were male and 41 (33.7%) females. Majority of the cases of PPHN, 78 (64.0%) were found to have gestational age above 37 weeks. Mode of delivery as cesarean section was noted in 70 (57.4%) cases while 52 (42.6%) had spontaneous vaginal delivery. Resuscitation was required among 93 (76.2%) cases.

**Table-I:** Characteristics of Newborns with PPHN.

Characteristics		Number (%)
Gender	Male	81 (66.3%)
	Female	41 (33.7%)
Gestational Age (weeks)	34-37	44 (36.0%)
	>37	78 (64.0%)
Maternal Hypertension		27 (22.1%)
Maternal Diabetes		12 (9.8%)
Mode of Delivery	Spontaneous Vaginal Delivery	52 (42.6%)
	Cesarean Section	70 (57.4%)
Need of Resuscitation	Not Needed	29 (23.8%)
	Oxygen Only	36 (29.5%)
	Positive Pressure Ventilation	57 (46.7%)
Outcome	Survived	96 (78.7%)
	Not Survived	26 (21.3%)

Diagnosis of PPHN was confirmed with the help of echocardiography among 108 (88.5%) of the cases while remaining 14 (11.5%) were diagnosed analyzing difference in pre-postductal SpO2 and/or PaO2 and/or positive hyperoxia-hyperventilation test.

Causes of PPHN among newborns studied are shown in Table-II. Meconium aspiration syndrome 52 (42.6%), birth asphyxia 48 (39.3%), respiratory distress syndrome 23 (18.8%) and sepsis 33 (27.0%) were found to be the commonest causes of PPHN. No cause of PPHN was identified among 18 (14.8%) cases.

**Table-II:** Postnatal Causes of PPHN among Newborns (n=122).

Causes of PPHN	Number (%)
Meconium Aspiration Syndrome	52 (42.6%)
Birth Asphyxia	48 (39.3%)
Respiratory Distress Syndrome	23 (18.8%)
Sepsis	33 (27.0%)
Congenital Pneumonia	9 (7.3%)
Lung Hypoplasia	6 (4.9%)
Idiopathic PPHN	18 (14.8%)

In terms of maximum respiratory support, majority, 81 (66.4%) newborns required continuous positive airway pressure while intermittent mandatory ventilation was administered in 23 (18.8%) and oxygen with mask in 18 (14.8%).

Severe PPHN was found to be the most frequent, noted among 63 (51.6%) while Moderate PPHN was observed in 40 (32.8%) and Mild PPHN in 19 (15.6%). Morality was noted among 26 (21.3%) of cases.

## DISCUSSION

PPHN progressing to respiratory failure has been documented from last five decades as it was initially noted by Gersony WM and coworkers in 1969.^12^ To the best of our knowledge, the current study is the largest single center study from Pakistan documenting causes and severity of PPHN.

In the present work, majority 81 (66.3%) of the cases having PPHN were male, 78 (64.0%) with gestational age above 37 weeks while 70 (57.4%) were having mode of delivery as cesarean section. Previous local findings have also witnessed male predominance among cases with PPHN while a recent multicenter study having six Asian countries including Pakistan also found that 61.2% of the cases having PPHN were male.^4^ Others have also labeled male gender to be more associated with PPHN.^5^,^7^ Cesarean section as mode of delivery is also found among majority of the cases in the past findings,^5^,^13^,^14^ which correlates with current findings. Others have also described increased chances of respiratory distress syndrome among PPHN cases with the presence of cesarean section as mode of delivery.^5^,^15^ Previous researchers have also found PPHN to be more common among post term newborns as was the case in the present finding.^7^,^9^ Harerimana I et al from South Africa^11^ noted 52.8% of their cases of PPHN to have born after 37^th^ week which is quite consistent to what we noted in the present finding. It is a well-established fact that during labor, higher levels of endogenous prostaglandins and catecholamines are released, that may along with physical compression attributed to birth canal, help in more clearance of lung fluid.^16^ The described process is hampered during cesarean section. This could be the reason that more cases of PPHN are found to have their mode of delivery as cesarean section.

In the current study, meconium aspiration syndrome 52 (42.6%), birth asphyxia 48 (39.3%), respiratory distress syndrome 23 (18.8%) and sepsis 33 (27.0%) were found to be the commonest causes of PPHN. Other researchers from Asian countries have shown the commonest etiology of PPHN to be meconium aspiration syndrome.^4^ Studies from Thailand and Australia also recorded more than half of the PPHN cases to have meconium aspiration syndrome.^17^-^19^ Data from Malaysia also noted 68.0% of their cases with PPHN to have meconium aspiration syndrome.^20^ Very similar to the present findings, Walsh-Sukys MC et al.^2^ also noted 41% of their PPHN cases to have meconium aspiration syndrome. Meconium causes mechanical blockage related to airways that can result in air trapping, hyper-inflation which can pose higher chances of pneumothorax. Meconium components have also been found to inactivate surfactant, triggering inflammatory process releasing cytokines, and can enhance the assembly of vasoconstrictors endothelin as well as thromboxane.^19^ Data from South Africa^11^ also found meconium aspiration syndrome (59.7%) to the main underlying pathology in PPHN cases. A local study by Khan I and Gul H from Lahore found that neonates with MAS had PPHN as most common complaint.^20^ Local data in the past have also pointed out birth asphyxia, sepsis and respiratory distress syndrome to be the commonest causes of PPHN which is very similar to the current findings.^5^ Steuer MA et al.^21^ from USA found sepsis to be the commonest etiology behind PPHN while we noted 27.0% of our cases to have sepsis. No cause of PPHN was identified among 18 (14.8%) cases which is very similar to what has been found recently in a multicenter trial from six Asian Countries where they found 12.5% cases of PPHN to have unknown etiology.^4^ PPHN is commonly secondary to pulmonary pathology but idiopathic PPHN is also noted among cases from other parts of the world.^1^,^8^

We noted severe PPHN was found to be the most frequent, noted among 63 (51.6%) while Moderate PPHN was observed in 40 (32.8%) and Mild PPHN in 19 (15.6%). These findings were in accordance to previously published data where 57% of the cases with PPHN were found to have severe PPHN while mild PPHN was seen in 29.1% and mild PPHN among 13.9% of the cases.^5^ Roofthooft MT and Colleagues^22^ from Netherland also noted 43.4% of their PPHN cases to have severe PPHN while remaining 56.6% were having mild to moderate PPHN. Another local study from Karachi,^23^ according to echocardiography, 17% of the cases with PPHN were found to have mild PPHN while 28% had moderate PPHN and 55% had severe PPHN which is again very similar to what was found in the present study.

This study gives us insight about the common causes and basic characteristics of cases reporting with PPHN. The current data must be considered a valuable addition to whatever little is known about different aspects of PPHN locally. More studies are needed to further explore the related aspects of PPHN among local population.

### Limitations of the study

We were unable to find out the current incidence of PPHN at our center. Also, we did not evaluate maternal or infant risk factors. No data regarding management or outcome of PPHN was included in the present study.

## CONCLUSION

Meconium aspiration syndrome, birth asphyxia and respiratory distress syndrome were noted to be the commonest postnatal causes of PPHN. Severe PPHN was found to be the most frequent form of PPHN.

### Authors’ Contribution:

**MSA:** Conceived, methodology, proof reading. Responsible for data’s authenticity and integrity.

**MA:** Literature Review, Introduction.

**HMA:** Drafting, data analysis.

**AZ:** Data Interpretation, Discussion.
